# Multidrug Resistance in Critically Ill Patients: An Unresolved Issue

**DOI:** 10.3390/microorganisms11040946

**Published:** 2023-04-04

**Authors:** Savino Spadaro

**Affiliations:** Intensive Care Unit, Department of Translational Medicine, University of Ferrara, 44121 Ferrara, Italy; spdsvn@unife.it

**Keywords:** multidrug resistance, sepsis, septic shock, hospital-acquired pneumonia (HAP), ventilator-associated pneumonia (VAP), critically ill patients

## Abstract

Sepsis and septic shock are common in critically ill patients and, as recommended by the Surviving Sepsis Campaign (SSC), early empiric antimicrobial therapy, specifically within the first hour, is crucial for the successful management of these conditions. To be effective, the antimicrobial therapy must also be appropriately administered: the drugs should cover the most probable pathogens and achieve effective concentrations at the site of infection. However, pharmacokinetics are frequently altered in critically ill patients and continuously change since the clinical conditions of these patients quickly and markedly change over time, either improving or deteriorating. Accordingly, optimizing antimicrobial drug dosing is fundamental in intensive care units (ICUs). This Special Issue of *Microorganisms* examines the epidemiology, diagnostic innovations, and strategies applied in the context of infections in critically ill patients with MDR infections.

## 1. Introduction

In recent years, there has been a notable increase in antimicrobial resistance and multi-drug resistance among critically ill patients. Several infections, such as hospital-acquired pneumonia (HAP) and ventilator-associated pneumonia (VAP), are the most common nosocomial infections in the intensive care unit (ICU) and are responsible for more than half of antibiotics prescribed in critical care settings. Despite efforts to improve an early diagnosis and management of infection, morbidity and mortality rates remain high, particularly if a multi-drug resistance (MDR) phenomenon occurs. Multiple factors contribute to determine the onset of MDR, so, for these reasons, an accurate and early identification of the responsible pathogen is required to implement the appropriate treatment that may impact the mortality.

## 2. Discussion

In this Issue, several interesting topics have been addressed regarding the diagnostic innovations and the strategies applied in the context of infections in critically ill patients.

Of particular interest is the overview on the management and diagnosis of nosocomial pneumonia offered by the review included in this Issue [1].

Diagnosing HAP or VAP remain a challenge at the bedside for physicians in ICUs. Despite the use of a chest X-ray being more common in ICUs, daily routine CXR is no longer recommended in ICU patients to assess the evolutive changes and the response to treatment. The use of lung ultrasound should be promoted as a tool for diagnosing HAP and VAP, particularly when combined with clinical information.

Nowadays, the traditional aetiologic diagnostic cultures approach fails to detect pathogens due to the administration of empirical antibiotics that require approximately from 48 h to 72 h to obtain definitive results from sample acquisition. New strategies need to be implemented in order to reduce the pathogen identification time and achieve the faster initiation of appropriate treatment or a faster switch to target treatment according to antimicrobial stewardship programs [1].

The advances in rapid molecular techniques for microbiological confirmation, such as rapid syndromic multiplex PCR tests have enhanced the diagnostic armamentarium of nosocomial infection. The introduction of the Syndromic Rapid Multi-Pathogen PCR panels has the advantage of identifying multiple targets from a sample in a timely manner. Of note, it allows for an early identification of MDR pathogens in order to facilitate enhanced infection control practices. The use of these advances for the early detection of serious bacterial infections will result in a decrease in antibiotic use and more targeted antimicrobial regimens. In addition, they have the potential to change the scene of the diagnostic and management approach of NP in the future, improving outcomes, as well as contributing to better antibiotic stewardship. However, further validations versus traditional diagnostic techniques are needed to verify their effects on antimicrobial prescribing, patient outcomes, and resistance.

During the last decade, new therapeutic options (beta-lactam/beta-lactamase inhibitor) have been approved for HAP and VAP, with positive results for several Gram-negative bacilli that represent a major problem, especially in the critical care setting. Beta-lactams are frequently administered in ICUs; they are one of the safest antibiotics but are not without side effects: neurotoxic manifestations occur in 10–15% of ICU patients and renal complications increase when they are associated with well-known nephrotoxic drugs such as vancomycin [2]. However, the clinicians should evaluate the benefit–risk ratio for the efficacy/toxicity of using antibiotics, especially in patients presenting pre-existing kidney disease, older patients, or patients with septic shock [2]. In an interesting review, Roger et al. [3], focusing on the misdiagnosis of beta-lactam-related adverse events, suggested the relevance of therapeutic drug monitoring and how to identify toxicity thresholds may help to better tailor beta-lactam dosing regimens in ICU patients. Several reports described the most common neurotoxic and nephrotoxic adverse events associated with the beta-lactam antibiotics routinely used in the ICU setting. According to this evidence, monitoring toxicity and antimicrobial resistance is indispensable to accept or refute the advantage of prolonged infusion of beta-lactam antibiotics in ICUs [3].

Ideally, individualized dosing strategies should account for the altered pharmacokinetic and pathogen susceptibility in each patient [4]. However, administering the appropriate therapy may be challenging in critically ill patients with infectious diseases, due to the dynamic and variable fluctuations. Fluid administration, alterations in protein-binding, and capillary permeability can affect the volume of distribution, while hemodynamic resuscitation may result in increased cardiac output and augmented renal clearance, with increased drug clearance and subsequent insufficient drug levels to treat less-susceptible strains [4]. Altered pharmacokinetics can result in insufficient B-lactams serum concentrations and can lead to treatment failure and increased selection pressure and resistance in critically ill patients.

From COVID-19, we learned that most bacterial infections (between 30% and 50%) were developed during ICU and hospital stay but the main mechanism involved in determining the occurrence of over infections remains unclear [5,6]. The reported incidence of MDR bacterial infections in critically ill COVID-19 patients is high; however, MDR infections are associated with a higher length of stay in ICU [5] and the acquisition rate of MDR in ICU patients appears similar during the first wave of COVID-19 compared to the previous period [7,8]. Interestingly, the analysis performed by Pasero et al. underlines that the extensive use of empirical broad-spectrum antimicrobial drugs and steroids treatment seem to play a role in developing secondary infections, especially of bacterial origin, with a variable rate of resistance [5]. In accordance with this lesson, the empiric antimicrobial therapy should be administered following an appropriate stewardship program that balances the effects and the risks of critically ill patients. 

Some critically ill patients require extracorporeal membrane oxygenation (ECMO) as cardio–pulmonary support in the case of life-threatening respiratory and/or cardiac failure. Since the use of ECMO is increasing in ICUs, it has become important to investigate whether this method is also able to modify the pharmacokinetics of different drugs. The impact of ECMO on the pharmacokinetics of some antimicrobial drugs frequently used in ICUs is discussed in this Special Issue [9,10].

## 3. Conclusions

In conclusion, many new drugs with broad spectrum activity against MDR pathogens represent promising options to enhance the antibiotic armamentarium in the clinical practice in ICUs. However, due to there being few data regarding the efficacy in practice, this require further investigation. The development of “new optimal dosing regimens”, based on pharmacokinetics/pharmacodynamic targets could guarantee clinical and microbiological efficacy, but pharmacokinetics /toxicodynamic targets could also reduce the risk of side effects. The use of dosing software may help to achieve these goals. Further research is needed to confirm that target attainment is better.

## Data Availability

Not applicable.
